# Case finding of Alpha-1 antitrypsin deficiency: never wasted time!

**DOI:** 10.1186/s40248-017-0115-2

**Published:** 2018-01-20

**Authors:** Bruno Balbi

**Affiliations:** Istituti Clinici Scientifici Maugeri IRCCS, Veruno, NO Italy

Alpha-1 Antitrypsin Deficiency (AATD) is considered the most important genetic cause of respiratory disorders, mainly COPD in its emphysematous phenotype [1, 2]. In high risk populations, i.e. those in which a high prevalence of AATD is suspected on the ground of previous data [3], up to 2% of the cases of COPD may, in fact, be due to AATD [4]. This prompted WHO to issue a recommendation that all COPD patients should be tested for AATD at least once in their life in order to ascertain if this genetic condition is underlying their disease [5] Much less strong recommendations are issued about a general population screening [5]. This is actually not recommended if not in high risk populations provided that a genetic counseling is offered to probands [3].

In the last decades many studies were published showing a worldwide distribution of AATD genes in basically the five continents and many regions and countries [6–9]. This type on analysis showed as expected large variations in the frequency of genes carrying the disorder across the different countries. Asia is not an exception to this finding. Allelic frequencies in India differ from those in other countries, e.g. Saudi Arabia. However, these data are often based on rather old studies, sometimes performed on a limited number of individuals.

In this issue of *Multidisciplinary Respiratory Medicine*, Zhumagaliyeva and Coll. report their data on a case-finding project they performed on a cohort of COPD patients in Kazakhistan [10]. This is the ninth largest country in the world, with an area of 2,724,900 km^2^ “(https://en.wikipedia.org/wiki/Kazakhstan)”.

Capitalizing on a European Respiratory Society Fellowship granted to the first Author of this paper, the study was generated by a collaboration with two of the most important AATD laboratories (the German and the Italian ones), Authors tested 187 patients with COPD and were able to determine the allelic frequencies for Z, S and I (rare) alleles in Kazakhistan. Interestingly, these frequencies were discrepant with those found in a previous, rather old, study performed in Kazakhistan [11]. This may be due to the more sensible and modern methodologies used in this study.

Overall, the results of this study confirm that AATD is present in Kazakhistan. Although the Authors did not find any patient with severe AATD (i.e. homozygous or double heterozygous for alleles carrying AATD), AATD alleles were identified and thus it is possible to estimate that subjects with AATD are in fact present among the population and even more among COPD patients in that country.

What is the lesson we can learn from the paper of Zhumagaliyeva and Coll? I think that fostering new studies on case finding for AATD in COPD is never a wasted effort. Identifying AATD genes in COPD patients may be relevant from many different points of view. Firstly, for the patient as this diagnosis may change entirely the therapeutic approach. Augmentation therapy (AT) with AAT extracted from pooled blood samples from blood donors is potentially available throughout the world and this can change the natural history of the disease. Recent evidences confirmed that AT can slow the loss of lung tissue and consequently of lung function in AATD COPD patients. In doing this AT may add years to the patients' life [12, 13]. Secondly, and not less important, it is the possibility of genetic testing for the patient’s family members, the so called non-index cases as opposed to the patient with diagnosed AATD, the index case. Testing may unveil new cases of AATD, usually diagnosed in less severe stages of the disease, leaving thus more space for secondary prevention and treatment. Last but not least, case-finding of AATD in COPD patients is a “must” in the third millennium medicine. Unvealing the genetic background should not be an episodic activity in modern medicine, especially when after a genetic diagnosis a disease-modifying treatment is potentially available.
